# Effects of Modified Attapulgite on Daily Weight Gain, Serum Indexes and Serum Metabolites in Fattening Beef Cattle

**DOI:** 10.3390/ani15152167

**Published:** 2025-07-23

**Authors:** Jiajie Wang, Hanfang Zeng, Hantong Weng, Haomiao Chang, Yunfei Zhai, Zhihui Huang, Chenchen Chu, Haihui Wang, Zhaoyu Han

**Affiliations:** 1 College of Animal Science and Technology, Nanjing Agricultural University, Nanjing 210095, China; 2023805109@stu.njau.edu.cn (J.W.); 2022105011@stu.njau.edu.cn (H.W.); 15117424@njau.edu.cn (H.C.); kint214@163.com (Y.Z.); 2023105008@stu.njau.edu.cn (Z.H.); 2023805110@stu.njau.edu.cn (C.C.); 2022105001@stu.njau.edu.cn (H.W.); 2 College of Animal Science and Technology, Gansu Agricultural University, Lanzhou 730070, China; 14236@gsau.edu.cn

**Keywords:** fattening beef cattle, modified attapulgite, antioxidant indicator, inflammatory factor, serum metabolism

## Abstract

In this study, we investigated the effects of dietary supplementation with thermally modified attapulgite on the daily weight gain, serum biochemical indices, and serum metabolites of Simmental fattening cattle. A total of 30 healthy Simmental fattening beef calves of similar age (8 to 9 months old) and body weight (370 ± 10 kg) were randomly divided into two groups, each containing 15 animals. A control group was fed the basal diet, and a treatment group was fed the same basal diet with the addition of 4 g/kg of thermally modified attapulgite. After 75 days of formal experiment, the calves in the two groups were weighed, and blood samples were collected by tail vein blood sampling for determinations of the serum biochemical indices and serum metabolites using liquid chromatography–mass spectrometry (LC-MS) analysis. The results indicated that the addition of thermally modified attapulgite to the diet had no significant effects on the daily weight gain of fattening beef cattle. After feeding with modified attapulgite, the glutathione peroxidase and superoxide dismutase activities in the serum of the experimental group were 55.02% (257.26 U·mL^−1^ to 165.95 U·mL^−1^, *p* < 0.05) and 13.11% (18.98 U·mL^−1^ to 16.78 U·mL^−1^, *p* < 0.05) higher than that in the control group. Compared with the control group, the tumor necrosis factor-alpha content was reduced by 14.50% (31.27 pg·mL^−1^ to 36.57 pg·mL^−1^, *p* < 0.01), and the concentration of interleukin-6 and lipopolysaccharide decreased by 17.00% (34.33 pg·mL^−1^ to 41.36 pg·mL^−1^, *p* < 0.001) and 23.05% (51.34 EU·L^−1^ to 66.72 EU·L^−1^, *p* < 0.001) in the serum of the experimental group. Contrastingly, the thermally modified attapulgite had no significant effects on the levels of serum total protein, albumin, or globulin in Simmental fattening cattle (*p* > 0.05). Furthermore, the results of serum metabolomic analyses revealed that there were a total of 98 differential metabolites, which were mainly enriched with respect to glycerophospholipid metabolism, Th1 and Th2 cell differentiation, autophagy-other, retrograde endogenous cannabinoid signaling, and the NF-κB signaling pathway. Overall, thermally modified attapulgite was found to effectively increase the activity of antioxidant enzymes, reduce serum inflammatory mediators, may suppress oxidative damage, enhance immunity, and have a positive influence on the health of Simmental fattening beef calves.

## Simple Summary

Many farms feed a large amount of concentrate every day in order to increase daily weight gain during the fattening stage of beef cattle, but too much concentrate will lead to a decrease in rumen pH, which will trigger a large number of Gram-negative bacteria to die and release lipopolysaccharide (LPS), which is not only easy to induce disease but also to exacerbate inflammation. The results of the present study suggest that adding 4 g/kg of modified attapulgite to the TMR can effectively increase the activity of antioxidant enzymes, reduce serum inflammatory mediators, may suppress oxidative damage, enhance immunity, and have a positive influence on the health of Simmental fattening beef calves.

## 1. Introduction

As a consequence of the progress in economic development in recent decades, there have been substantial increases in living standards worldwide, among the corollaries of which have been an increase in the demand for beef. This trend accordingly necessitates a transition from beef cattle farming to a more scientific and efficient model. On many farms, during the fattening stage, beef cattle are currently fed hay supplemented with a large number of concentrates in order to promote increases in daily weight gain, and, consequently, the feed concentrate-to-roughage ratio is generally high. However, an excess of concentrate can lead to reductions in rumen pH, which may have adverse effects on the gut populations of Gram-negative bacteria. Upon degradation, the cells of dead bacteria release lipopolysaccharides (LPSs), which, when absorbed by the host organism, may induce a range of diseases and, in severe cases, even cause death [1]. In addition, it has been shown that during the inflammatory response to accumulated LPS, these compounds induce phagocytosis via the specific receptor, which in turn promote the secretion of inflammatory factors such as TNF-α and IL-6, thereby further exacerbating the inflammatory response [2,3,4,5,6]. This will not only affect the feed intake of beef cattle, but will also lead to changes in the body’s metabolism [7], where more nutrients will be directed to support the immune response process [8].

The findings of numerous studies have, however, provided evidence to indicate that feed additives can contribute positively to enhancing animal immunity [9], production performance [10], antioxidant capacity [11], and anti-inflammatory responses [12]. Some studies have shown that the addition of astragalus polysaccharides to diets can regulate energy metabolism through glucose metabolism and amino acid metabolism in heat-stressed cows [13]. Moreover, feeding beef cattle with oregano essential oil has been demonstrated to improve meat quality and antioxidant capacity [14,15], and in dairy cows, was found to be effective in reducing somatic cell counts and had favorable anti-inflammatory effects, thereby contributing to the control of subclinical bovine mastitis caused by *Staphylococcus aureus* and *E. coli*. These findings accordingly indicate that suitable feed additive supplementation can play a key role in promoting the health of livestock [16,17]. In view of the above challenges, the search for a substance that can remove endotoxins and improve the anti-inflammatory ability of beef cattle is now of great importance for beef cattle production.

The thermally modified attapulgite (referred to as modified attapulgite) was used in this experiment, which was made from natural attapulgite by roasting at 500 °C and then crushed and sieved through a 200-mesh sieve. Studies have shown that after natural bentonite is treated at 500 °C, its cation exchange capacity (CEC) increases by 22.53 mmol/100 g, and its specific surface area increases by 15.3 m^2^/g [18]. Its unique layered structure can form a cover on the surface of the intestinal mucosa, thereby reducing the direct contact between harmful substances and intestinal epithelial cells [19], thus facilitating the absorptive elimination of these substances via the feces, and hence improving the intestinal environment and reducing the levels of endotoxins and inflammatory mediators in the blood [20,21]. Previous studies in this regard have shown that supplementation of the feed of dairy cows with 10 g/kg of natural attapulgite can contribute to increases in milk yield and the protein content of milk and reduce the microbial content of milk [22]. In addition, the large surface area-to-volume ratio of attapulgite facilitates the adsorption of large amounts of ammonia and water, thereby retarding the decomposition of organic matter, and by adding to padding as deodorant and desiccant, can also contribute to enhancing the growth environment of dairy cows [23,24]. To date, however, there has been comparatively little research devoted to assessing the effects of dietary supplementation with modified attapulgite in the rearing of fattening beef cattle. Accordingly, in this study, we sought to investigate the effects of dietary modified attapulgite on the daily weight gain, serum biochemical indices, and serum metabolites of Simmental fattening beef cattle by adding 4 g/kg of modified attapulgite to TMR, with the aim of providing a theoretical basis for the elimination of serum endotoxins and inflammatory factors, as well as for assessing the application of modified attapulgite in cattle production.

## 2. Materials and Methods

The experimental design and procedures were performed in accordance with the Institutional Animal Care and Use Committee of the Nanjing Agricultural University, China (SYXK2011-0036).

Animal experiments. This study was conducted from March to June 2024 at the Taierlan Beef Cattle Farm in Shenyang City, Liaoning Province, China. As experimental animals, we selected 30 Simmental calves of similar age (8 to 9 months old) and body weight (370 ± 10 kg) during the fattening period, which were randomly allocated to one of two groups (experimental and control), each comprising 15 calves. The modified attapulgite used in this study, which was thermally modified from natural attapulgite by roasting at 500 °C and then crushed and sieved through a 200-mesh sieve, was provided by Jiangsu Sinitic Biological Technology Co., Ltd. in Xuyi, China. The main components of modified attapulgite are 52.30% SiO_2_, 12.29% Al_2_O_3_, 5.67% MgO, 8.65% Fe_2_O_3_, 2.59% CaO, 2.38% K_2_O, and 0.18% Na_2_O. The purity of the modified attapulgite in the fed material is 56%.

In this experiment, drawing on relevant feeding trials such as those of Wang [25], Yang [26], and Lian [27], it was finally decided that the experimental group would be supplemented with total mixed ration with 4 g/kg of modified attapulgite. The concentrate formulation and other formulations of TMR were the same for beef cattle in the control and experimental groups, and the cattle were stanchion-fed twice daily (at 05:00 and 15:00) with free access to water. The experimental period was divided into a 7-day pre-test period and a 75-day main test period. The nutrient levels in the total mixed ration provided in this study were established with reference to the Beef Cattle Feeding Standard (NY/T 815-2004) [28]. The composition and nutrient components of the experimental ration are shown in Table 1.

Determination of daily weight gain. On the 7th day of the pre-experimental period and 75th day of the main trial period, 30 beef calves were weighed, with the total weight gain of each cow being divided by the number of days of the trial to determine the daily weight gain.

Measurement of serum indicators. At 08:00 on the 75th day of the main experimental period, 10 mL of blood was collected from the tail vein of beef calves in each group. The samples were transferred to a coagulation tube and left to stand for 1 h, then centrifuged at 1900× *g* for 10 min to separate the serum, which was transferred to a 1.5 mL centrifuge tube, and stored at −80 °C for subsequent analyses (Centrifuge model: DLAB Scientific Co., Ltd., Beijing, China; #DM0412). We monitored total protein (TP), albumin (ALB), globulin (GLB, by calculation), superoxide dismutase (SOD), glutathione peroxidase (GSH-Px), and malondialdehyde (MDA) using the microplate method. Respective test kits were purchased from the Nanjing Jiancheng Bioengineering Institute (Reagent kit model: Nanjing, China; #A045-4, #A028-2-1, #A001-3, #A005-1, and #A003-1, respectively). These were analyzed by using a Microplate Reader (Spark, TECAN, Männedorf, Switzerland). The levels of interleukin-6 (IL-6), tumor necrosis factor-alpha (TNF-α), and lipopolysaccharide (LPS) were determined by ELISA. Respective test kits were purchased from Nanjing MALLBIO Biological Technology Co., Ltd. (Nanjing, China; #MBE10288, #MBE10037, #MBE11054). These indexes were detected by using the Microplate Reader (RT-6100, Rayto, Shenzhen, China).

Measurement of serum metabolites. To characterize the non-targeted metabolome in the serum collected from cattle, we performed liquid chromatography–mass spectrometry (LC-MS), the data of which were subsequently analyzed.

Sample preparation for LC-MS analysis [29,30]. Serum samples maintained at −80 °C were thawed at 4 °C and vortexed-mixed for 1 min. Aliquots (100 μL) of the serum were added to a 1.5 mL centrifuge tube along with 400 μL of an acetonitrile: methanol = 1:1 (*v*:*v*) solution containing 0.02 mg·mL^−1^ internal standard (L-2-chlorophenylalanine) for the extraction of metabolites. The samples were mixed by vortexing for 30 s and thereafter sonicated at low temperature for 30 min (5 °C, 40 KHz). The sonicates thus obtained were subsequently placed at −20 °C for 30 min to precipitate the proteins, prior to centrifugation for 15 min at 13,000× *g* and 4 °C. The resulting supernatants were collected and blown dry under nitrogen, prior to re-solubilization with 100 µL of acetonitrile: water = 1:1 and extraction using low-temperature ultrasonication for 5 min (5 °C, 40 KHz). Following subsequent centrifugation for 10 min at 13,000× *g* and 4 °C, the supernatants were transferred to sample vials for LC-MS/MS analysis. The LC-MS/MS analysis of the sample was conducted on a Thermo UHPLC-Exploris480 (Thermo Fisher Scientific Inc., Waltham, MA, USA) system equipped with an ACQUITY HSS T3 column (100 mm × 2.1 mm i.d., 1.8 μm; Waters, Milford, MA, USA) at Majorbio Bio-Pharm Technology Co., Ltd. (Shanghai, China).

Liquid chromatography conditions [29]. Samples for analysis (10 μL) were separated on an HSST3 column (100 mm × 2.1 mm i.d., 1.8 µm, Waters, Milford, MA, USA) and passed into the mass spectrometric detector. Mobile phase A was a water:acetonitrile (95:5, *v*/*v*) solution containing 0.1% formic acid and mobile phase B was an acetonitrile:isopropanol:water (47.5:47.5:5.0, *v*/*v*/*v*) solution containing 0.1% formic acid. The gradient elution program for positive and negative ion modes is shown in Table 2. The flow rate was 0.40 mL·min^−1^ and the column temperature was 40 °C.

Mass spectrometry conditions [29,31]. Mass spectrometric data were collected using a Thermo UHPLC-Exploris480 Mass Spectrometer equipped with an electrospray ionization (ESI) (Thermo Fisher Scientific Inc., Waltham, MA, USA) source operating in positive and negative ion modes. The optimal conditions were set as follows: Aux gas heating temperature: 400 °C; capillary temperature: 350 °C; sheath gas flow rate: 50 psi; Aux gas flow rate: 15 psi; ion-spray voltage floating: −2800 V in negative mode and 3000 V in positive mode; and normalized collision energy: 20-40-60 eV rolling for MS/MS. The full MS resolution was 60,000, and the MS/MS resolution was 15,000. Data acquisition was performed using the Data Dependent Acquisition (DDA) mode.

## 3. Statistical Analyses

Each group consisted of 15 sample data, with a total of 30 sample data across both groups. The data obtained from the serum biochemical tests were initially entered into Excel 2016, and subsequently analyzed using a one-way ANOVA using SPSS 26.0 software to determine the differences between groups. Data are expressed as the means ± standard error, with *p*-values of >0.05, <0.05, <0.01, and 0.001 being designated as insignificant, significant, very significant, and extremely significant, respectively. Metabolites exhibiting VIP values greater than 1.0 and *p*-values from two-tailed Student’s *t*-tests less than 0.05 were classified as differential metabolites [29,32].

For serum metabolomics, we used the R package “ropls” (version 1.6.2) to perform principal component analysis (PCA) and orthogonal least partial squares discriminant analysis (OPLS-DA), and seven-cycle interactive validation was performed to evaluate the stability of the model. Metabolites with a variable importance in projection (VIP)-value > 1 and *p*-value < 0.05 were designated as significantly different metabolites based on the VIP values obtained using the OPLS-DA model and *p*-values obtained using Student’s t-test [29,33]. Differential metabolites were identified based on metabolic pathway annotation using the KEGG database for pathways involving the differential metabolites. The Python package scipy.stats (Version 1.0.0) was used to perform pathway enrichment analysis and Fisher’s exact test was used to determine the biological pathways most relevant to experimental treatments [32].

## 4. Results

### 4.1. Effects of Modified Attapulgite on the Daily Weight Gain of Simmental Fattening Cattle

As shown in Table 3, compared with the control group cattle, there were no significant differences (*p* > 0.05) in daily weight gain of cattle in the experimental group.

### 4.2. Effects of Modified Attapulgite on Serum Indices in Simmental Fattening Cattle

Among the cattle fed a diet supplemented with modified attapulgite, we detected that the GSH-Px and SOD activities in the serum of the experimental group increased by 55.02% (257.26 U·mL^−1^ to 165.95 U·mL^−1^, *p* < 0.05), 13.11% (18.98 U·mL^−1^ to 16.78 U·mL^−1^, *p* < 0.05) compared to the control group. The TNF-α content in the experimental group was reduced by 14.50% (31.27 pg·mL^−1^ to 36.57 pg·mL^−1^, *p* < 0.01), the concentration of IL-6, LPS decreased by 17.00% (34.33 pg·mL^−1^ to 41.36 pg·mL^−1^, *p* < 0.001), and 23.05% (51.34 EU·L^−1^ to 66.72 EU·L^−1^, *p* < 0.001), respectively (Table 4). Contrastingly, supplementation with modified attapulgite had no significant effects (*p* > 0.05) on the serum levels of TP, ALB, or GLB in Simmental fattening cattle, although we detected a non-significant tendency of increase in TP (*p* > 0.05).

### 4.3. Effects of Modified Attapulgite on Serum Metabolomics in Simmental Fattening Cattle

**Figure 1:**
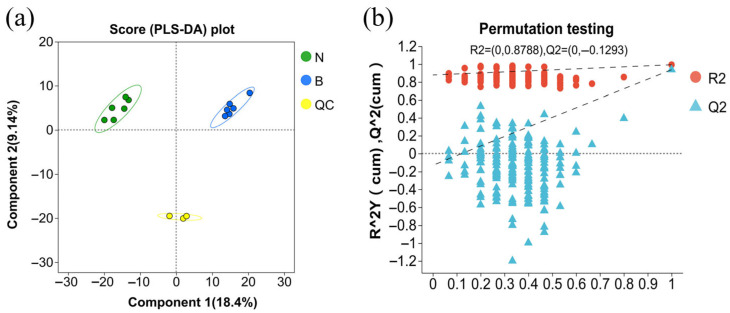
Serum PLS-DA score (**a**) and PLS-DA model validation (**b**) plots. Note: N: experimental group serum samples, B: control group serum samples, QC: quality control samples, R2: model interpretability, Q2: model predictability. The closer the R2 and Q2 values are to 1, the more stable and reliable is the model.

Partial least squares discriminant analysis. To assess differences in the metabolite profiles of cattle in the control and experimental groups, we performed partial least squares discriminant analysis (PLS-DA), using which, partial least squares regression was undertaken to establish a regression model between metabolite expression and the sample category to obtain a prediction of sample category. As shown in the serum PLS-DA score plot presented in Figure 1a, samples from the experimental and control cattle were widely separated, thereby indicating a significant categorization effect, and thus implying that feeding modified attapulgite had a notable effect on the serum metabolism of Simmental fattening beef cattle. As shown in Figure 1b, values of Q2 and R2 were close to 1, with the value R2 being larger than that of Q2. In addition, the intercept of the regression line of Q2 with the Y-axis was found to be less than 0, thereby indicating that the model was not overfitted and was accurate and credible, and thus could be could be used for subsequent analyses.

**Figure 2:**
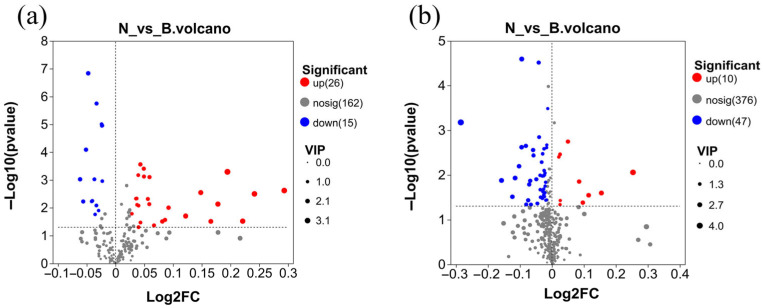
Volcano plots of serum differential metabolites in anion (**a**) and cation (**b**) modes. Note: N: serum samples from the experimental group, B: serum samples from the control group.

Screening for differential serum metabolites. In total, we identified 98 differential metabolites (based on a VIP > 1 and *t*-test *p* < 0.05 as threshold criteria for differential expression), with significant changes in the experimental group compared with the control group, among which, there were 26 significantly upregulated and 15 significantly downregulated metabolites in the anionic mode (Figure 2a) and 10 significantly upregulated and 47 significantly downregulated metabolites when analyzed in the cationic mode (Figure 2b).

**Figure 3:**
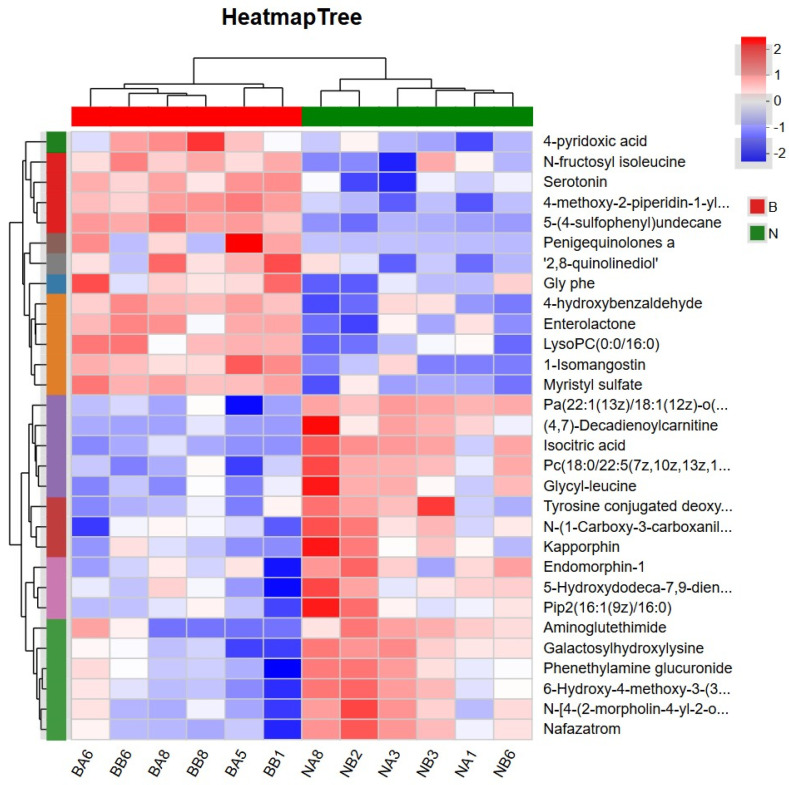
Hierarchical clustering heat map of differential metabolites. Note: N: experimental group serum samples, B: control group serum samples. The red and blue colors indicate up- and downregulated expression of the differential metabolites, respectively.

Cluster analysis of differential metabolites. On the basis of the VIP > 1 and *p* < 0.05 thresholds, we clustered and analyzed the top 30 differential metabolites. As shown in Figure 3, samples from the experimental and control group cattle were clustered into two distinct categories, thereby indicating clear differences between the groups, and that samples from the two groups could be differentiated based on their differential metabolite profiles. Moreover, we detected no evident differences between the six samples within the groups, thus confirming the reliability of the PLS-DA model in differentiating between the different groups. Importantly, these findings provide evidence that feeding Simmental fattening cattle a diet supplemented with modified attapulgite has a notable effect on the production of metabolites detected in the serum of these cattle.

**Figure 4:**
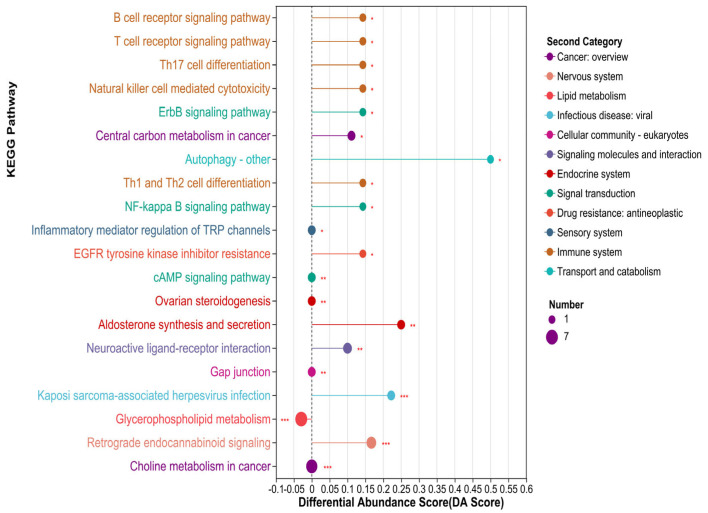
A plot showing differential abundance scores. Note: The size of the dots indicates the number of differentially annotated metabolites in a pathway. The longer the line segment with the dots to the right of the central axis, the more upregulated is the overall expression of the pathway, whereas the longer the line segment with the dots to the left of the central axis, the more downregulated is the overall expression of the pathway. *** *p*-value < 0.001, ** *p*-value < 0.01, * *p*-value or < 0.05.

KEGG pathway analysis of differential metabolites. On the basis of our KEGG pathway analysis, we detected the enrichment of 98 differential metabolites in 54 metabolic pathways (*p* < 0.05) (Supplementary Figure S1). Among these, analysis of the top 20 KEGG metabolic pathways revealed that differential metabolites in experimental and control cattle were mainly enriched in glycerophospholipid metabolism, Th1 and Th2 cell differentiation, autophagy-other, retrograde endocannabinoid signaling, the NF-κB signaling pathway, and other metabolic pathways (Figure 4). Differential abundance scores revealed that the overall expression of most of these metabolic pathways was upregulated, with only the glycerophospholipid metabolic pathway identified as being downregulated overall. There is one differential metabolite involved in the autophagy-other forms of metabolic pathway, four differential metabolites involved in the retrograde endocannabinoid signaling metabolic pathway, one differential metabolite involved in the NF-kB signaling pathway, one differential metabolite involved in the Th1 and Th2 cell differentiation metabolic pathway, and six differential metabolites involved in the metabolic pathway involved in glycerolphospholipid metabolism (Figure 4 and Table 5).

## 5. Discussion

Previous studies have evaluated the effect of attapulgite as a non-nutritive feed additive on daily weight gain in ruminants. Some studies have shown that adding 2 g/kg of attapulgite to the diet of Holstein calves had no significant effect on daily weight gain [34]. When montmorillonite was added to the TMR fed to Hu sheep, there was no significant change in daily weight gain [35], which is consistent with the results of this experiment. This also demonstrates that adding this material to cattle diets does not adversely affect daily weight gain, possibly because modified montmorillonite is not a nutritional additive, and therefore does not influence daily weight gain.

Levels of serum TP, a sum of ALB and GLB, serve as an important indicator of protein metabolism in the body. ALB is synthesized by hepatocytes, the content of which can indicate the synthetic function of the liver, which is an important source of protein for the body [36], and is also involved in processes such as the maintenance of blood osmolality and fatty acid transport [37], whereas globulins play key immunity-related roles [38]. Yang et al. [26] found that modified attapulgite had no significant effect on TP, GLB, and ALB in the serum of dairy cows. Similarly, Lian et al. [27] reported that feeding modified attapulgite to dairy cows during pre-lactation significantly increased total serum protein, but the changes in GLB and ALB content were not significant. In the present study, we found that dietary supplementation with modified attapulgite tended to promote an increase in TP, although the differences were not significant, and, similarly, we detected no significant changes in the levels of ALB and GLB, thereby tending to indicate that modified attapulgite would not markedly influence the protein metabolism of Simmental fattening cattle. We speculate that the main reason for this is that modified attapulgite has a small effect on the liver and does not affect its ability to synthesize proteins [39].

It is generally believed that SOD activity levels indirectly reflect the body’s ability to scavenge oxygen free radicals, whereas the levels of MDA provide an indirect measure of the severity of cell damage caused by the attack of free radicals [40]. GSH-Px also has the capacity to scavenge free radicals, thereby contributing to the protection of cell membranes and preventing necrotic cell death [41]. In this study, we found that dietary supplementation with modified attapulgite promoted significant increases in the activities of serum SOD and GSH-Px, thus indicating that this material could contribute to enhancing serum antioxidant capacity in cattle. Consistently, Lin et al. [42] have found that attapulgite promoted significant increases in GSH-Px activity and reductions in MDA content in dairy cows. In this regard, Pavelić et al. [43] have suggested that such improvements in oxidative status may be attributable to the adsorptive role of clay minerals, such as modified attapulgite, which function as scavengers of reactive oxygen species, thereby reducing the amount of reactive oxygen in the blood. Alternatively, these authors speculate that the effects of this material could be associated with increases in the levels of GSH-Px, and hence enhanced the levels of free radical scavenging.

TNF-α is primarily produced by macrophages and lymphocytes and plays important roles in processes of cell proliferation and apoptosis, inflammatory response, and immune regulation. Accordingly, the status immune system activation and the intensity of immune responses in organisms can be assessed via a determination of the levels of TNF-α [44]. IL-6 secreted by Th2-type cytokines is associated with an exacerbation of inflammatory responses [45], and it has been established that the long-term sustained release of IL-6 contributes to reductions in immune function and metabolic dysregulation, along with tissue and organ damage [46]. The NF-κB signaling pathway is a classic inflammatory signaling pathway that participates in the regulation of various cytokines and mediates the body’s inflammatory response, thereby initiating the expression of target genes such as TNF-α and IL-6 [47]. It has also been demonstrated that LPS, as an important stimulator of inflammatory responses in vitro and in vivo, is cytotoxic to mammals. LPS can activate the binding of NF-κB to Toll-like receptor 4 (TLR4), thereby activating the downstream NF-κB pathway, and thus inducing and exacerbating inflammatory responses [48,49]. In this study, we detected a significant reduction in the levels of TNF-α in cattle fed a modified attapulgite-supplemented diet, along with highly significant reductions in the levels of LPS and IL-6, and a significant upregulation of the NF-κB signaling pathway. In this regard, we speculate that in response to a reduction in LPS levels, there was a reduction in the activation of NF-κB signaling pathway proteins, thereby blocking the downstream NF-κB pathway, and thus contributing to reductions in the levels of TNF-α and IL-6. Moreover, we suspect that these effects may be mediated via genes associated with the NF-κB signaling pathway, including MyD88, TLR-2, TLR-4, TLR-9, TRAF-6, TNF-α, and IL-6 [50]. It is also conceivable that an increase in the expression of other genes in the pathway and a reduction in the expression of TNF-α and IL-6 genes, or the upregulation of other pathways with anti-inflammatory functions offset some of the effects, leading to an overall upregulation of the pathway and a reduction in TNF-α and IL-6 levels, although the specific underlying mechanisms need to be further investigated.

The endogenous cannabinoid system in animals, which includes arachidonoylethanolamine and 2-arachidonylglycerol ether [51], plays important roles in disorders associated with lipid metabolism and neurological dysfunction. These cannabinoids have been established to have important antioxidant, anti-inflammatory, and inhibitory effects, including a suppression of the release of TNF-α [52]. In this study, we detected significant reductions in the levels of TNF-α and IL-6 in the serum of the experimental group cattle, in which we also detected an upregulated expression of the retrograde endogenous cannabinoid signaling pathway. Accordingly, on the basis of these findings, we infer that by mediating retrograde endogenous cannabinoid signaling, supplementary modified attapulgite may have contributed to an inhibition of the release of TNF-α and IL-6. This may be due to the modified attapulgite’s large specific surface area and strong adsorption capacity, which prolongs the retention time of feed in the digestive tract and improves the digestion and absorption rate of feed nutrients [53], and nutrients such as diglycerides are more easily absorbed by the gastrointestinal tract in these circumstances, which in turn promotes the upregulation of the expression of retrograde endocannabinoid signaling pathways [54]. We argue, based on Iuvone’s hypothesis [55], that upregulation of this pathway leads to upregulation of cannabinoid 2 (CB2), which inhibits the release of pro-inflammatory mediators from mast cells by binding to receptors on mast cells and sensitizing these cells to the anti-inflammatory effects exerted by endogenous cannabinoids.

Autophagy, a type II programmed cell death, is an important cellular process which has been established to be activated during inflammation and development. It not only inhibits the activation of inflammatory vesicles, but also the expression of the TLR4/MyD88 (myeloid differentiation factor 88) pathway, whereas a reduction in the release of TNF-α and IL-6 has been found to be associated with a silencing of TLR4 and MyD88, thereby influencing the progression of inflammation [56,57]. On the basis of our findings of reduced TNF-α and IL-6 levels and an upregulated expression of the autophagy pathway in cattle fed a modified attapulgite-supplemented diet, we speculate that the observed reductions in serum inflammatory factors might also be associated with an activation of autophagy, and that autophagy could contribute to controlling the secretion of inflammatory factors by inhibiting the expression of TLR4 and MyD88, and the activation of inflammatory vesicles [58,59,60].

The sole downregulated pathway identified in this study is the glycerophospholipid metabolism pathway. In the membranes of mammalian cells, phosphatidylcholine (Pc) and phosphatidylethanolamine (Pe) are the most abundant phospholipids. Therefore, oxidative damage caused by cell membrane disruption is closely related to abnormal glycerophospholipid catabolism [61]. Some inflammatory factors such as TNF-α can activate phospholipase A2 (PLA2), and after PLA2 activation, PC is cleaved to obtain lysophosphatidylcholine (LPC) [62], which is the main product of glycerophospholipid metabolism [63]. LPC is a major constituent of oxidatively damaged low-density lipoproteins (oxLDL) that induce lymphocyte and macrophage migration and increase the production of pro-inflammatory cytokines, as well as promote oxidative stress and apoptosis, thereby exacerbating inflammation and contributing to disease development [62]. In this study, we detected reductions in the levels of LPC, which may be due to the reduction in inflammatory factors (e.g., TNF-α, IL-6) in vivo after feeding modified attapulgite inhibits the activation of PLA2 [64], which reduces the rate of PC hydrolysis to generate LPC, further reducing the production of inflammatory factors and reducing oxidative damage.

Th1 cells secrete cytokines such as IL-2, IFN-γ, and TNF-α that function in cellular immunity and delayed hypersensitivity inflammatory responses, and play an important role in autoimmune diseases and host resistance to intracellular pathogen infections [65,66]. Th2 cells contribute to the differentiation of B cells into antibody-secreting cells and play important roles in humoral immune responses, as well as in allergic and infectious diseases [67,68,69]. The transcription factor T-bet, encoded by the TBX21 gene, serves as a specific marker for cytokine secretion by Th1-type cells, and GATA-3 has been identified as the only transcription factor regulating Th2-type cytokine synthesis [70]. The findings of previous studies have indicated that by regulating Th1 and Th2 cell differentiation, the expression of the TBX21 and Gata-3 genes influences the secretion of cytokine [71,72]. The upregulation of the Th1 and Th2 cell differentiation pathway in this experiment, with the potential to enhance immunocompetence, may be due to the increased uptake of glycerol diesters by the modified attapulgite [54], as shown in Table 5. Glycerol diesters as the main differential metabolites of the Th1 and Th2 cell differentiation pathway, affected the upregulation of the pathway, but whether this was caused by upregulation of the expression of the TBX21 and Gata-3 genes needs to be further explored.

## 6. Conclusions

Collectively, our findings in this study revealed that basal dietary supplementation with 4 g/kg of thermally modified attapulgite is effective in promoting increases in the activities of antioxidant enzymes and reducing the levels of serum inflammatory mediators. Consequently, as a supplement, we believe that this material has potential utility in retarding oxidative damage in beef cattle, as well as in enhancing immune system function, thereby contributing to improvements in the health of beef cattle.

## Figures and Tables

**Table 1:** Composition and nutrient content of the experimental diet (dry matter basis).

Diet Composition	Content, % of Diets
Corn	44.66
Salt	0.89
Baking soda	0.89
Magnesium oxide	0.07
Calcium carbonate	1.23
Corn germ meal	9.44
Soybean meal	9.42
Palm kernel powder	2.44
Chicken bone meal	1.48
Rice straw	20.72
Distillers Dried Grains with Solubles	8.76
Total	100.00
Nutrient component of TMR	Content, % of DM
Crude ash	11.33
Ether extract	2.99
Crude protein	14.42
Calcium	1.08
Phosphorus	0.30
Neutral detergent fiber	34.37
Acid detergent fiber	17.96

**Table 2:** Gradient separation procedure for positive and negative ion modes.

A		B	
Time, Min	Mobile Phase, %	Time, Min	Mobile Phase, %
0~3	0~20	0~1.5	0~5
3~4.5	20~35	1.5~2	5~10
4.5~5	35~100	2~4.5	10~30
5~6.3	100	4.5~5	30~100
6.3~6.4	100~0	5~6.3	100
6.4~8	0	6.3~6.4	100~0
		6.4~8	0

Note: A and B are positive and negative ion mode elution procedures, respectively.

**Table 3:** Effect of modified attapulgite on the daily weight gain of Simmental fattening cattle.

Item	Control Group	Modified Attapulgite	*p*_Value
Initial weight, kg	377 ± 6.95	365 ± 5.10	0.204
Final weight, kg	507 ± 8.63	499 ± 8.81	0.525
Average daily weight gain, kg/d	1.75 ± 0.08	1.79 ± 0.11	0.752

**Table 4:** Effect of modified attapulgite on serum indexes of Simmental fattening cattle.

Item	Control Group	Modified Attapulgite	*p*_Value
TP, µg·mL^−1^	66.54 ± 2.12	71.08 ± 1.59	0.097
ALB, µg·mL^−1^	44.33 ± 1.85	40.87 ± 2.84	0.316
GLB, µg·mL^−1^	23.76 ± 2.63	31.00 ± 3.79	0.13
GSH-Px, U·mL^−1^	166 ± 21.61	257 ± 22.47	0.012
MDA, nmol·mL^−1^	2.61 ± 0.51	2.95 ± 0.41	0.62
SOD, U·mL^−1^	16.78 ± 0.67	18.98 ± 0.74	0.035
IL-6, pg·mL^−1^	41.36 ± 0.93	34.33 ± 0.74	<0.001
TNF-α, pg·mL^−1^	36.57 ± 1.29	31.27 ± 0.92	0.003
LPS, EU·L^−1^	66.72 ± 2.06	51.34 ± 1.148	<0.001

**Table 5:** Metabolic pathways and related differential metabolites.

Metabolic Pathways	*p*_Value	up\down	Related Differential Metabolites (up\down)	VIP_Value
Th1 and Th2 cell differentiation	0.017	up	DG (20:4 (8z, 11z, 14z, 17z)/18:0/0:0) (up)	1.251
Retrograde endocannabinoid signaling	0.007	up	Pc (18:0/22:5 (7z, 10z, 13z, 16z, 19z)) (up)	1.042
			DG (20:4 (8z, 11z, 14z, 17z)/18:0/0:0) (up)	1.251
			Pc (18:3 (9z, 12z, 15z)/16:0) (up)	1.451
			Pe (36:2) (up)	1.284
Autophagy-other	0.017	up	Pe (36:2) (up)	1.284
NF-kappaB signaling pathway	0.017	up	Dg (20:4 (8z, 11z, 14z, 17z)/18:0/0:0) (up)	1.253
Glycerophospholipid metabolism	0.0002	down	Pc (18:0/22:5 (7z, 10z, 13z, 16z, 19z)) (up)	1.984
			LPC (18:3 (6z, 9z, 12z)/0:0) (down)	1.517
			PG (i-12:0/a-17:0) (down)	1.398
			PC (18:3 (9z, 12z, 15z)/16:0) (up)	1.451
			LPC (20:5 (5z, 8z, 11z, 14z, 17z)/0:0) (down)	1.889
			LPC (17:0/0:0) (down)	1.157

Note: VIP: variable importance in projection.

## Data Availability

The original contributions presented in this study are included in the article/Supplementary Materials. Further inquiries can be directed to the corresponding author.

## Supplementary Materials

The following supporting information can be downloaded at: https://www.mdpi.com/article/10.3390/ani15152167/s1. Figure S1: KEGG pathway enrichment analysis.
