# Supplementary material for: Effects of Modified Attapulgite on Daily Weight Gain, Serum Indexes and Serum Metabolites in Fattening Beef Cattle
Source: Animals (Basel). 2025 Jul 23;15(15):2167. doi: 10.3390/ani15152167 (PMC12345580; doi:10.3390/ani15152167)
Supplement: Supplementary file 1 [file animals-15-02167-s001.zip › animals-3716807-supplementary.pdf]

Comparison of test data for thermally modified attapulgite

Natural attapulgite, is a hydrous magnesium-aluminum silicate mineral. Its modification by different environments can greatly enhance certain properties and increase its functionality.

The main method of thermal modification is roasting, which can increase the specific surface area of Aconite, enhance the active centers and adsorption sites, and improve its adsorption properties.

The effect of thermal modification temperature on adsorption：

Adsorption of aflatoxin by different mineral sources at different temperatures

Natural attapulgite	40℃	100℃	200℃	300℃	500℃
Decolorization rate 21%	53. 23%	69. 60%	66. 23%	87. 00%	90. 68%
Decolorization rate 25%	59. 12%	60. 17%	68. 54%	88. 81%	100. 00%
Decolorization rate 34%	59. 10%	67. 99%	77. 87%	76. 54%	100. 00%
Decolorization rate 59.11%	45. 19%	49. 42%	53. 06%	76. 22%	100. 00%
Decolorization rate 63.05%	42. 62%	44. 73%	50. 94%	75. 28%	93. 84%
Decolorization rate 69.22%	86. 28%	81. 84%	88. 81%	100. 00%	100. 00%

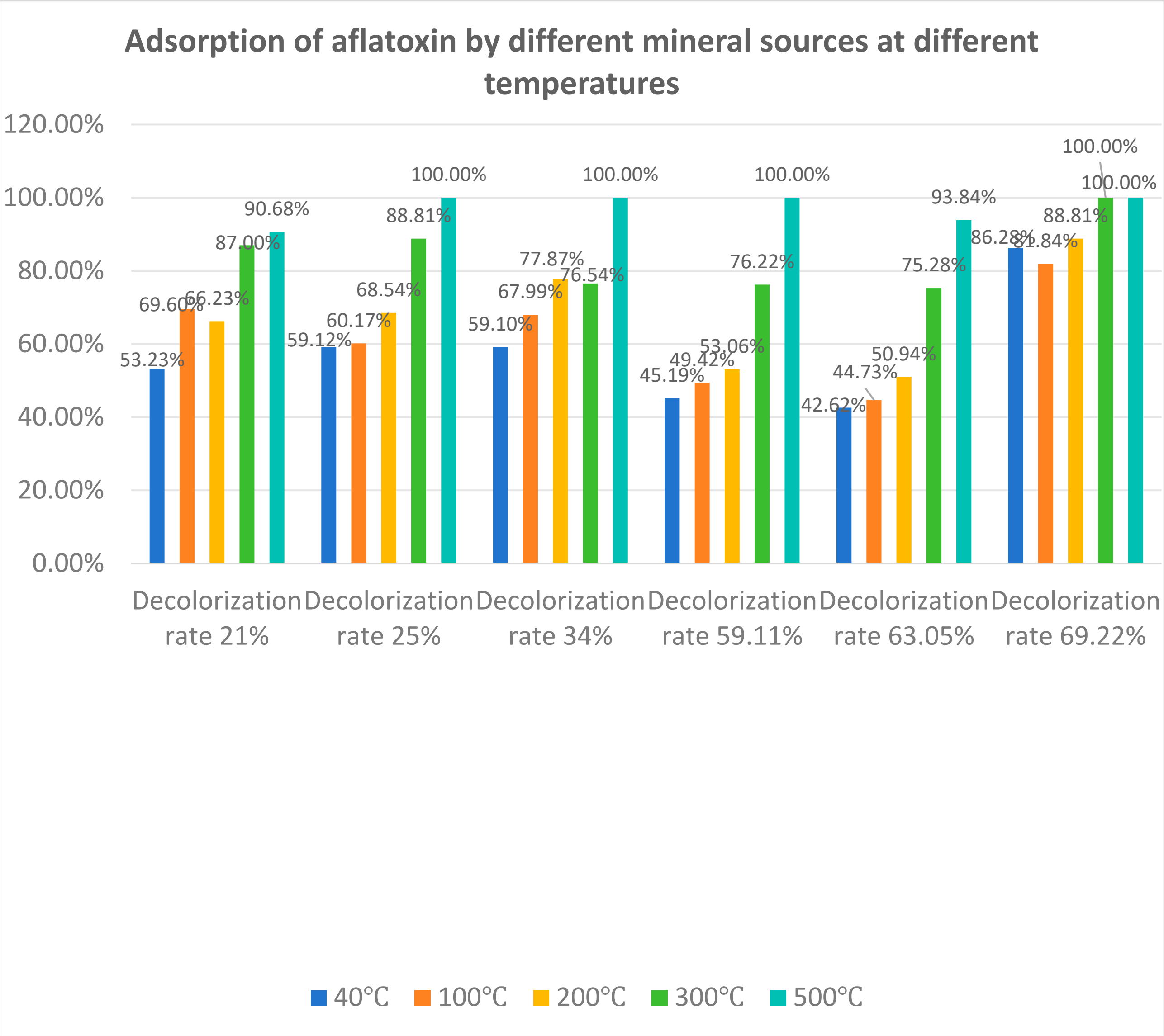

Effect of thermal modification on specific surface area:

Effect of Temperature on Contrasting Surfaces

Natural attapulgite	100℃	200℃	300℃	500℃	600℃
52. 08	53. 24	56. 12	135. 12	140. 21	80. 56

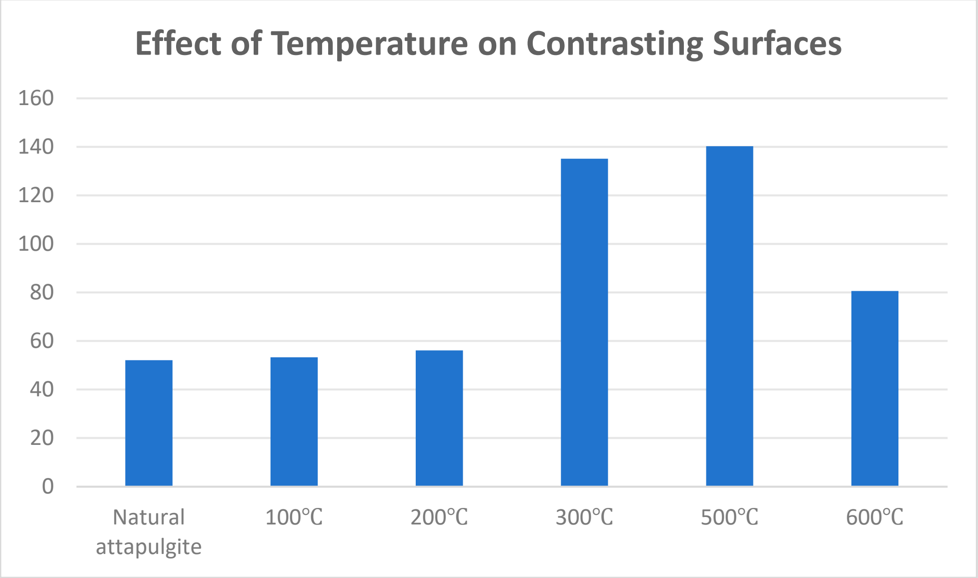

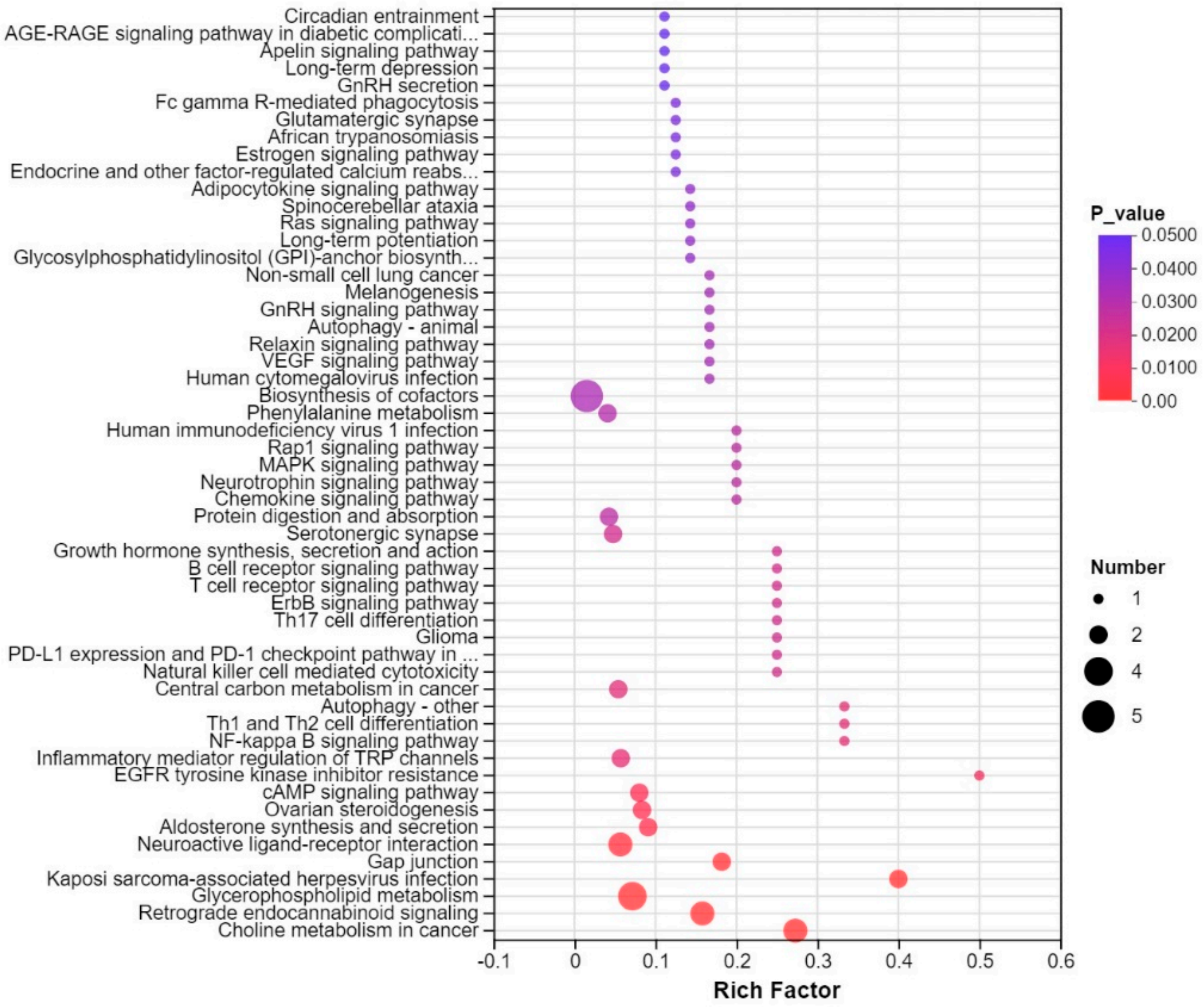

Figure S1. KEGG pathway enrichment analysis. Note: The size of the dots represents the number of differential metabolites enriched in the pathway, and a redder dot color indicates greater significance.
